# Achados incidentais de aneurismas torácicos e abdominais

**DOI:** 10.1590/1677-5449.007616

**Published:** 2016

**Authors:** Adenauer Marinho de Oliveira Góes, Bárbara Íris Mascarenhas, Sofia Cunha Rodrigues, Mariseth Carvalho de Andrade, Reinaldo Sergio Monteiro Franco

**Affiliations:** 1 Universidade Federal do Pará – UFPA, Faculdade de Medicina, Belém, PA, Brasil.; 2 Centro Universitário do Estado do Pará – CESUPA, Belém, PA, Brasil.; 3 Universidade do Estado do Pará – UEPA, Belém, PA, Brasil.

**Keywords:** aneurisma, dilatação patológica, ectasia, achados incidentais

## Abstract

**Contexto:**

Os aneurismas de aorta abdominal (AAAs) são os mais comuns. A incidência anual de ruptura do AAA é de oito casos por 100.000 habitantes. A detecção incidental pode beneficiar o paciente, desde que o diâmetro seja monitorado e o paciente receba o tratamento adequado.

**Objetivos:**

Estimar a prevalência do diagnóstico incidental de aneurisma de aorta torácica (AAT) e de AAA em tomografias computadorizadas (TCs); avaliar a prevalência de sexo e idade dos pacientes e determinar quais as artérias acometidas e as características morfológicas dos aneurismas; determinar quais as indicações de TC mais associadas ao diagnóstico incidental de aneurismas.

**Métodos:**

Estudo descritivo, retrospectivo e randomizado. Critérios de inclusão: pacientes com 50 anos ou mais submetidos a TC de tórax, abdome ou pelve. Critérios de exclusão: acompanhamento ou suspeita diagnóstica de aneurismas. Foram utilizados protocolos com questões sobre dados demográficos e anatômicos.

**Resultados:**

Foram analisados 1.202 laudos radiológicos. Detectados 27 aneurismas (prevalência de 2,2%). Pacientes: 60% do sexo masculino e 40% do sexo feminino (p < 0,05). Localização: 13 casos (48,2%) na aorta ascendente (AAT); 7 (25,9%) na aorta infrarrenal (AAA); 2 (7,4%) aorta na transição toracoabdominal (ATA); 2 (7,4%) na ilíaca comum; 1 (3,7%) na ilíaca interna; 1 (3,7%) na artéria esplênica; e 1 (3,7%) na artéria renal.

**Conclusões:**

A maioria dos pacientes foi do sexo masculino (60%); houve maior frequência de AAT (diâmetro médio de 4,1 cm), seguido de AAA (diâmetro médio de 4,0 cm) e ATA (diâmetro médio de 3,9 cm). A principal indicação para a realização de TC associada ao diagnóstico incidental de aneurismas foi em função de sintomas respiratórios.

## INTRODUÇÃO

Define-se aneurisma como a dilatação arterial maior que 50% em relação ao diâmetro normal para o segmento em questão^1^^-^ 20. De acordo com a literatura, é mais frequente na aorta, seguida das artérias ilíacas e depois da esplênica^7^. Tem etiologia multifatorial - hereditária, traumática, infecciosa, inflamatória -, mas cerca de 80% dos aneurismas estão associados à degeneração aterosclerótica da parede arterial^2^^,^ 5.

Estima-se que a incidência do aneurisma de aorta torácica (AAT) seja de seis casos por 100.000 pacientes/ano, e a do aneurisma de aorta abdominal (AAA), de 25 por 100.000 pacientes/ano^3^^,^ 5. Cerca de 10% dos pacientes com AAA têm aneurismas em outro segmento da aorta, e em 25% dos portadores de AAT, há um AAA concomitante^3^^,^ 7. O tratamento eletivo do AAT é recomendado quando o mesmo é sintomático ou quando atinge 6 cm de diâmetro^1^^,^ 8 ^,^ 20.

Com o aumento da expectativa de vida, o diagnóstico de AAA tem sido mais frequente; na população acima de 65 anos, chega a 6%, e acima de 80 anos, é de 10%^21^. O diagnóstico é mais frequente entre 65 e 75 anos^2^^,^ 9 ^,^ 10 ^,^ 22. A maioria dos trabalhos considera AAA infrarrenal uma dilatação com diâmetro transverso maior ou igual a 3 cm^9^^,^ 11 ^,^ 12 ^,^ 19 ^,^ 20 ^,^ 23.

O tratamento eletivo do AAA é recomendado quando o diâmetro atinge 5,5 cm ou quando há aumento de pelo menos 5 mm em 1 ano, devido ao aumento do risco de ruptura e da potencial letalidade.

O aneurisma isolado das ilíacas (sem comprometimento aórtico) é raro. Consideram-se aneurismáticas as artérias ilíacas comum e externa com diâmetro transverso igual ou maior a 1,5 cm. A abordagem eletiva é indicada para os aneurismas sintomáticos e para aqueles maiores que 2,5 cm^9^^,^ 12 ^,^ 23 ^,^ 24. Aneurismas de ilíaca interna devem ser tratados independentemente do diâmetro^20^.

O aneurisma de esplênica é o terceiro mais frequente entre os intra-abdominais e o mais comum entre os viscerais (46 a 60%); é o mais comum em mulheres (75 a 87%) com idade entre 50 e 79 anos^25^^,^ 26. Indicações terapêuticas incluem diâmetro superior a 2 cm, mulher em idade fértil, gestação antes do terceiro trimestre, se associados a pancreatite ou pseudocisto pancreático ou em caso de sintomas^20^^,^ 21 ^,^ 25 ^-^ 28.

Os aneurismas apresentam manifestações clínicas variáveis. Frequentemente, são assintomáticos e podem ser detectados incidentalmente ou por programas de rastreamento^3^^,^ 4 ^,^ 14 ^,^ 15 ^,^ 29.

O diagnóstico incidental é o achado radiológico inesperado em exames realizados com outra finalidade; ocorre em 5 a 20% dos exames radiológicos^30^^,^ 31. O diagnóstico incidental de aneurismas tem se tornado mais frequente devido ao aumento da expectativa de vida da população, ao refinamento de métodos diagnósticos e à maior facilidade de acesso aos mesmos^5^^,^ 9 ^,^ 16 ^,^ 31.

Estima-se que a incidência anual de ruptura do AAA seja de oito casos por 100.000 habitantes, sendo responsável por 2% das mortes na população acima de 60 anos^9^.

O tratamento eletivo dos aneurismas apresenta menor morbimortalidade; o diagnóstico incidental e os programas de rastreamento podem contribuir para a diminuição da mortalidade e de complicações associadas à doença aneurismática^9^^,^ 13 ^,^ 29 ^,^ 31 ^,^ 32.

## OBJETIVOS

Objetivo geral:

- Estimar a prevalência do diagnóstico incidental de AATs e AAAs em um serviço privado de radiologia.

Objetivos específicos:

Determinar:

- Sexo e idade dos pacientes com diagnósticos incidentais de AATs e AAAs.
- As artérias acometidas e as características morfológicas dos aneurismas incidentalmente diagnosticados.
- As indicações de realização de tomografias mais associadas ao diagnóstico incidental de AATs e AAAs.

## MÉTODOS

Foi realizado um estudo transversal, descritivo, retrospectivo, randomizado e com cálculo de tamanho amostral. Os dados foram coletados a partir de laudos de tomografias computadorizadas (TCs) de pelve, abdome e tórax realizadas em um serviço privado entre janeiro de 2009 e dezembro de 2013. A pesquisa foi aprovada pelo Comitê de Ética em Pesquisa da instituição e registrada na Plataforma Brasil.

Aplicou-se um protocolo de pesquisa com questões sobre: sexo do paciente, idade, data do exame, uso de contraste iodado endovenoso, diâmetro, comprimento, localização e forma (sacular ou fusiforme) do aneurisma. Os termos “aneurisma”, “dilatação”, “dissecção” e “ectasia” foram pesquisados nos laudos randomizados para a amostra.

Foram considerados aneurismas da aorta as dilatações com diâmetro maior ou igual a 3 cm, e o diâmetro adotado para classificação das ilíacas como aneurismáticas foi igual ou maior que 1,5 cm, conforme metodologias adotadas em trabalhos anteriores na literatura^9^^,^ 11 ^,^ 12 ^,^ 23. Dilatações menores foram classificadas como ectasias. Dilatações detectadas em outras artérias foram classificadas de acordo com descrições na literatura.

Para o cálculo do tamanho amostral, foi utilizada fórmula segundo a distribuição normal, com intervalo de confiança de 95%. A randomização foi realizada através do Programa BioEstat 5.0.

Os critérios de inclusão foram: TCs de tórax, abdome e/ou pelve de pacientes com 50 anos ou mais realizadas entre janeiro de 2009 e dezembro de 2013. Critérios de exclusão: TC solicitada por suspeita diagnóstica ou para acompanhamento de aneurismas previamente diagnosticados.

Foram utilizados os testes G de aderência e qui-quadrado (χ2). O nível de significância adotado foi de α = 0,05 (5%).

## RESULTADOS

Entre janeiro de 2009 e dezembro de 2013, foram realizadas 3.325 TCs de tórax, 3.132 TCs de abdome e 692 TCs de pelve em pacientes que atenderam aos critérios de inclusão do estudo. Após o cálculo de tamanho amostral e a randomização, foram selecionados 1.202 laudos radiológicos.

Foram examinados 343 (28,5%) laudos de TC de tórax, 612 (51%) de abdome, 247 (20,5%) de pelve. A TC de abdome foi estatisticamente mais frequente na amostra (p < 0,0001, teste χ2) (Tabela 1).

**Tabela 1:** Distribuição das tomografias quanto à topografia.

**Topografia**	**Frequência**	**%**
Abdome*	612	51%
Pelve	247	20,5%
Tórax	343	28,5%
TOTAL	1.202	100%

* p < 0,0001 (qui-quadrado).

O uso de contraste iodado endovenoso de acordo com a topografia da TC está descrito na Tabela 2. No tórax, os exames sem contraste foram estatisticamente mais frequentes; no abdome, os exames com contraste foram mais comuns (p < 0,0001, teste χ2); na pelve, não houve diferença significativa.

**Tabela 2:** Distribuição das tomografias quanto à topografia e ao uso de contraste iodado endovenoso.

**Topografia**	**Uso de contraste**				***p-valor***
	**SIM**	**%**	**NÃO**	**%**	
Abdome	415	67,8%	197	32,2%	< 0,0001*
Pelve	127	51,4%	120	48,6%	0,6554
Tórax	102	29,7%	241	70,3%	< 0,0001*
TOTAL	644	53,6%	558	46,4%	------

* p < 0,05.

**Figura 1:**
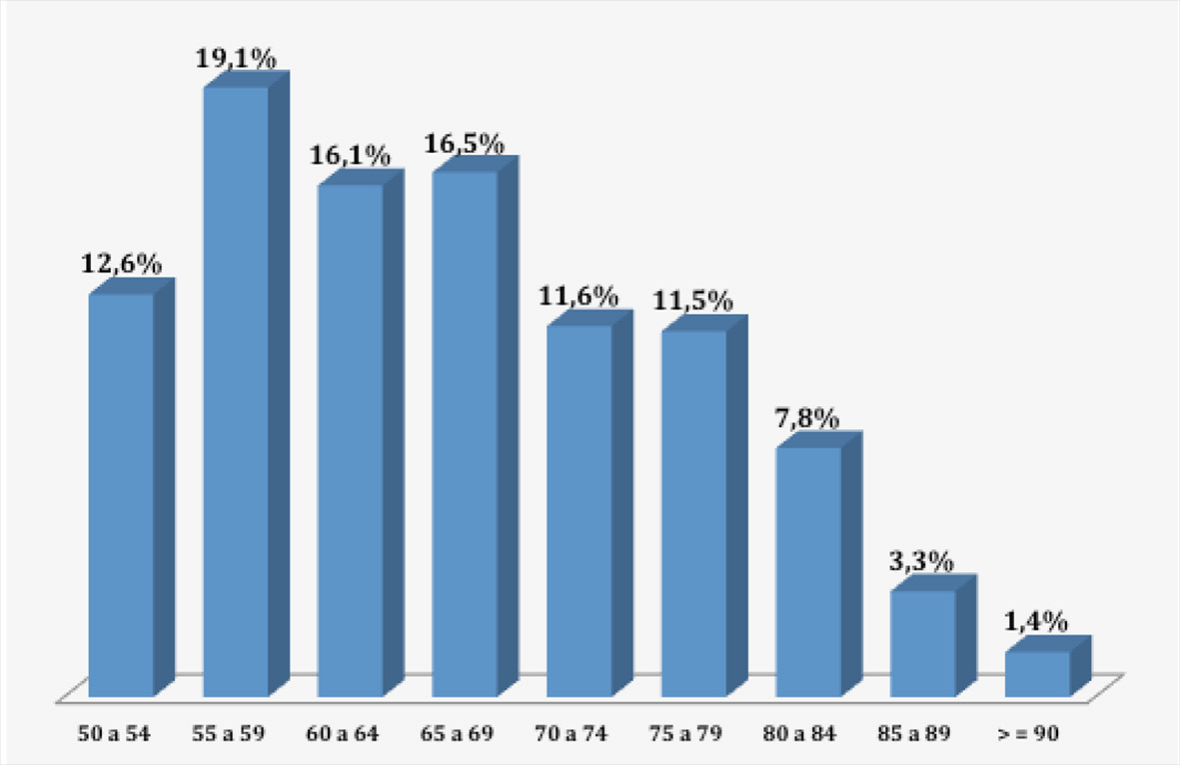
Distribuição quanto à faixa etária da amostra. Fonte: Banco de dados utilizado na pesquisa.

A idade dos pacientes submetidos aos exames variou de 50 e 96 anos, com média de 66,4 anos. Em relação ao gênero, 729 (60,6%) exames foram realizados em pacientes do sexo feminino e 473 (39,4%) no sexo masculino (p < 0,05, teste χ2) (Figuras 1 e 2).

**Figura 2:**
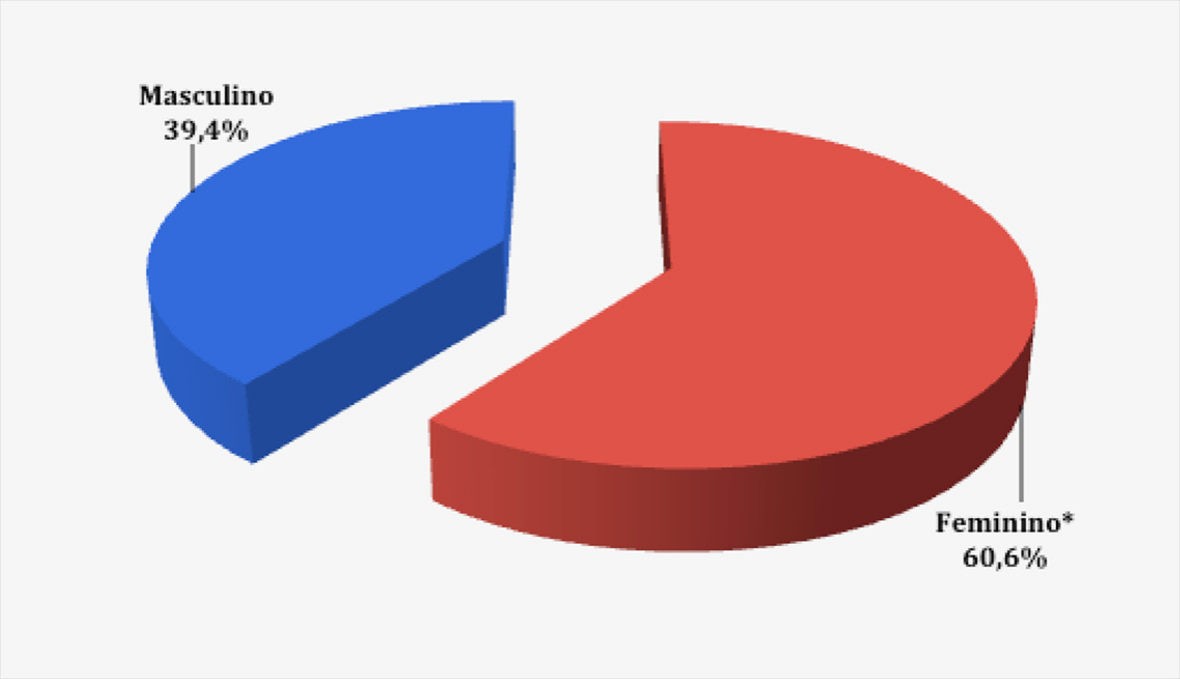
Distribuição da amostra quanto ao gênero. *p < 0,05.

**Figura 3:**
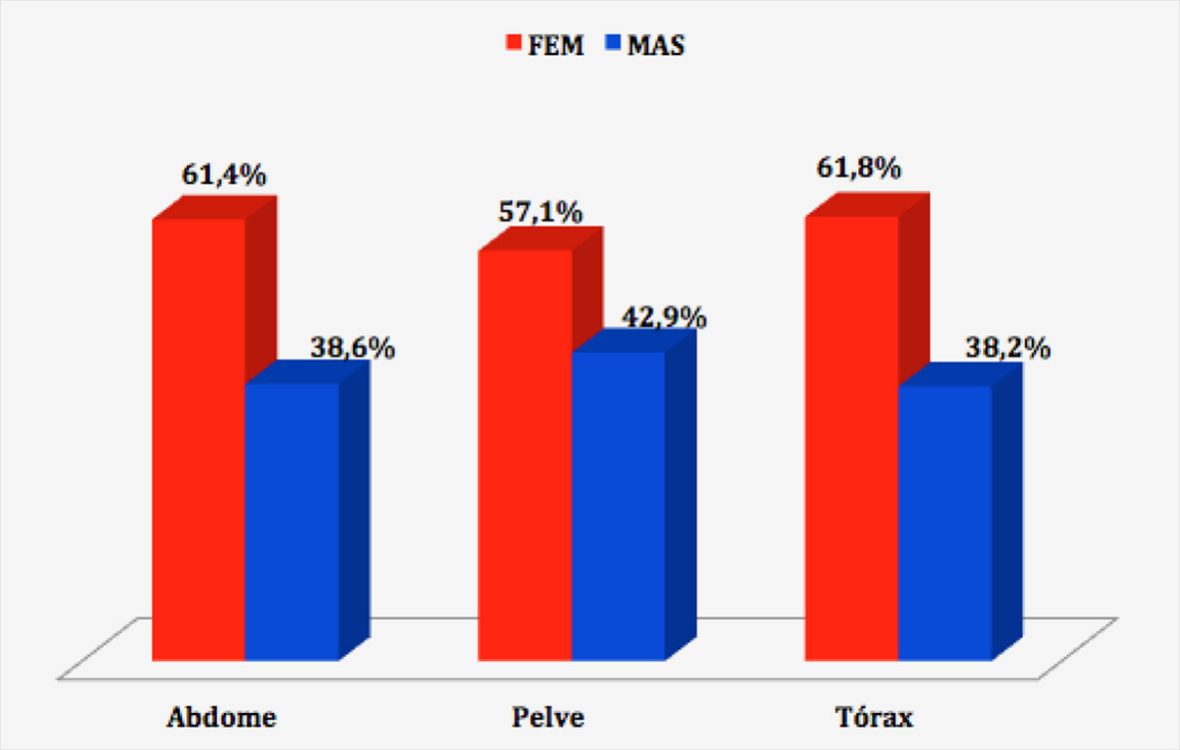
Distribuição quanto à topografia do exame e ao gênero dos pacientes.

A distribuição do gênero dos pacientes de acordo com a topografia do exame não demonstrou diferença estatisticamente significativa (p = 0,4674, teste χ2) (Figura 3).

A avaliação da topografia das TCs quanto à idade dos pacientes mostrou que a faixa etária entre 50 e 59 anos foi estatisticamente mais frequente entre os pacientes que realizaram TC de abdome; já a significância estatística nas TCs de tórax e pelve ocorreu na faixa etária de 60 a 69 (p < 0,0001, teste χ2) (Tabela 3).

**Tabela 3:** Distribuição quanto à topografia da TC e à faixa etária dos pacientes.

**Faixa etária (em anos)**	**Topografia**					
	**Abdome**	**%**	**Pelve**	**%**	**Tórax**	**%**
50 a 59	214*	35%*	84	34%	84	24,5%
60 a 69	181	29,6%	90*	36,4%*	120*	35%*
70 a 79	141	23%	52	21,1%	85	24,8%
80 a 89	68	11,1%	19	7,7%	47	13,7%
> = 90	8	1,3%	2	0,8%	7	2%
TOTAL	612	51%	247	20,5%	343	28,5%
p-valor	< 0,0001*		< 0,0001*		0,0010*	

* p < 0,0001* (qui-quadrado).

Houve 27 diagnósticos incidentais de aneurisma, sendo 15 (55,5%) encontrados entre as 343 TCs de tórax, 7 (26%) entre as 612 TCs de abdome e 5 (18,5%) entre as 247 TCs de pelve. O diagnóstico incidental de aneurismas foi estatisticamente mais frequente em TCs de tórax na comparação com as demais topografias (p = 0,0446, teste χ2) (Tabela 4).

**Tabela 4:** Distribuição dos aneurismas incidentalmente diagnosticados de acordo com a topografia da TC.

**TOPOGRAFIA**	**n**	**Achados**	**%**
Tórax	343	15*	4,4%*
Pelve	247	5	2%
Abdome	612	7	1,1%

* p = 0,0446 (qui-quadrado).

Os 27 aneurismas acometeram 25 pacientes (dois pacientes do sexo masculino apresentaram dois aneurismas simultaneamente). A distribuição dos aneurismas de acordo com a artéria acometida, o diâmetro médio do aneurisma e o gênero e a idade média dos pacientes está apresentada na Tabela 5. Entre os pacientes portadores de aneurismas de aorta infrarrenal, o sexo masculino foi estatisticamente mais frequente (p = 0,0116, teste G de aderência).

**Tabela 5:** Distribuição dos aneurismas quanto localização, gênero, idade e diâmetro médio.

**Localização**	**n**	**FEM**		**MAS**		**Idade média**	**Diâmetro médio**	**Teste G de aderência** **p-valor**
Aorta ascendente	13	7	53,8%	6	46,2%	71,9	4,1	0,844
Aorta na transição toracoabdominal	2	1	50%	1	50%	72,5	3,9	N/A
Aorta abdominal infrarrenal	7	2	28,6%	5	71,4%*	77	4	0,0116*
Artéria ilíaca comum direita	1	0	0%	1	100%	66	2,6	N/A
Artéria ilíaca comum esquerda	1	0	0%	1	100%	76	3	N/A
Artéria ilíaca interna esquerda	1	0	0%	1	100%	85	1,7	N/A
Artéria renal esquerda	1	0	0%	1	100%	89	2,2	N/A
Esplênica	1	0	0%	1	100%	62	1,2	N/A

* p < 0,05 (teste G de aderência).

N/A: não se aplica.

Indicações de realização das tomografias: as indicações por quadros respiratórios foram estatisticamente mais frequentes (p = 0,0001, teste G de aderência) e corresponderam a 10 casos. As demais indicações estão resumidas na Tabela 6.

**Tabela 6:** Distribuição quanto à indicação para realização das tomografias computadorizadas em pacientes com diagnóstico incidental de aneurisma.

**Indicação médica**	**Frequência**	**%**
Sintomas respiratórios	10*	37%*
Sintomas geniturinários	5	18,5%
Sintomas gastrointestinais	5	18,5%
Sintomas gerais	3	11,1%
Tumor hepático	2	7,4%
Não relatada	2	7,4%
TOTAL	27	92,6%

* p = 0,0001 (teste G de aderência).

Entre outros achados dignos de nota, foram encontradas sete ectasias arteriais, que acometeram com maior frequência a aorta abdominal, e o fato de que, entre os 25 pacientes diagnosticados com aneurismas arteriais, 20 apresentavam placas ateromatosas na aorta.

## DISCUSSÃO

As referências na literatura sobre diagnóstico incidental de aneurismas habitualmente enfocam uma determinada artéria; na maioria das vezes, a aorta abdominal. Este trabalho difere dos demais ao pesquisar o diagnóstico incidental de aneurismas acometendo qualquer artéria torácica e/ou abdominal; no entanto, os resultados foram estratificados de modo que pudessem ser comparados com os da literatura.

A predominância da doença aneurismática em homens já foi descrita por vários autores^1^^-^ 17 ^,^ 22 ^,^ 23 ^,^ 30 ^-^ 32. Apesar da amostra ser composta por 60,6% de TCs de pacientes do sexo feminino, houve uma predominância (embora sem diferença estatisticamente significativa) de diagnósticos no sexo masculino [15 (60%) homens e 10 (40%) mulheres].

De acordo com a literatura, o diagnóstico de AAA é mais frequente entre 65 e 75 anos^9^^,^ 11 ^,^ 12 ^,^ 23. Entre os portadores de AAA, a idade média foi 77 anos, semelhante à apontada por Barros et al. em um estudo de rastreamento de AAA na população de Vitória, que foi de 74,1 anos^16^; e superior à detectada por Silva et al., em que a média foi de 69,4 anos^10^.

A prevalência geral dos aneurismas arteriais foi de 2,2%. O AAA teve prevalência de 0,6% se analisados ambos os gêneros e de 1% quando considerados apenas os homens. A prevalência de AAA detectada foi inferior à relatada por Molnar et al.^12^ (2,1%), Barros et al.^16^ (2,5%), Bonamingo e Siqueira^11^ (3,7%) e Silva et al.^10^ (4,49%). O diagnóstico de AAA foi mais frequente em pacientes do sexo masculino (71,4%), concordando com a literatura^1^^-^ 9 ^,^ 17 ^,^ 22 ^,^ 30 ^-^ 32.

O diâmetro médio dos AAAs foi de 4 cm (variando entre 3,1 cm e 6,9 cm), aproximando-se de dois estudos brasileiros, os quais detectaram diâmetros médios de 3,9 cm^9^ e 3,5 cm^16^.

O tratamento eletivo do AAA é recomendável quando seu diâmetro atinge 5,5 cm^4^^,^ 11 ^,^ 13 ^,^ 32. Um dos AAAs incidentalmente diagnosticados apresentava diâmetro de 6,9 cm. Destacamos esse achado pois estima-se que o risco de rotura em 5 anos para aneurismas com diâmetro menor que 5 cm seja inferior a 5%, enquanto diâmetros superiores a 5 cm acarretam 25 a 43% de risco^1^^,^ 3.

A prevalência de AATs foi de 0,99%. Os 13 AATs encontrados acometeram a aorta ascendente, segmento já descrito como o mais acometido entre os da aorta torácica^3^. A dilatação progressiva da aorta ascendente pode levar à insuficiência valvar aórtica, à dissecção aguda ou à ruptura espontânea^1^, eventos que alteram dramaticamente a sobrevida dos pacientes.

Também foram encontrados dois aneurismas na transição toracoabdominal da aorta, o que representa 7,7% do total de aneurismas e uma prevalência de 0,16% na amostra. Não encontramos trabalhos sobre diagnóstico incidental de aneurismas nessa topografia. Porém, há referências de que aneurismas da transição toracoabdominal representam 10,6^3^ a 31,6%^5^ entre todos os aneurismas aórticos.

No presente estudo, houve predomínio de AATs, que representaram praticamente metade de todos os aneurismas encontrados, apesar da maioria das TCs avaliadas ter sido de abdome (50,9%). Esse resultado discorda de pesquisas anteriores, que afirmam ser infrarrenal o segmento aórtico mais acometido pelos aneurismas^1^^,^ 3 ^,^ 8 ^,^ 10 ^,^ 13.

O diâmetro médio dos AATs foi de 4,1 cm; entretanto, devido à carência de literatura sobre o tema, esse resultado não pôde ser comparado; o mesmo ocorreu com a média de idade dos pacientes portadores de AAT. Nenhum paciente apresentou diâmetro maior ou igual a 6 cm, a partir do qual a intervenção cirúrgica habitualmente é indicada^1^^,^ 8.

Os aneurismas de artéria ilíaca comum são candidatos à cirurgia eletiva quando seus diâmetros ultrapassam 2,5 cm^12^^,^ 23 ^,^ 24, e os da ilíaca interna, com qualquer diâmetro^20^. Foram encontrados dois aneurismas de artéria ilíaca comum, um com 3,0 cm e outro com 2,6 cm de diâmetro, além de um aneurisma de artéria ilíaca interna medindo 1,7 cm.

De acordo com as referências, em 85% dos aneurismas de artéria ilíaca comum há associação com AAA; essa associação é de 10% para aneurismas de artéria ilíaca interna e 1% para aneurismas de artéria ilíaca externa^24^. O único caso de aneurisma de artéria ilíaca interna detectado em nossa amostra apresentou AAA concomitante. Apesar de a literatura referir associação frequente entre os aneurismas de artérias ilíacas comuns e os da aorta infrarrenal^20^^,^ 23 ^,^ 24 ^,^ 33, neste trabalho, os casos de ilíaca comum ocorreram de modo isolado.

De acordo com a literatura, aneurismas viscerais predominam no sexo feminino e apresentam as seguintes indicações de tratamento cirúrgico: diâmetro maior ou igual a 2 cm, ruptura, sintomas, crescimento rapidamente progressivo, principalmente em gestantes ou em mulheres em idade fértil^20^^,^ 25. Os aneurismas viscerais mais comuns são: artéria esplênica (46 a 60%), artérias renais (17 a 30%), hepática (9 a 20%), mesentérica superior (3 a 5%) e tronco celíaco (3 a 4%)^20^^,^ 25. Nesta casuística, foi detectado um aneurisma de artéria esplênica com diâmetro de 1,2 cm em um paciente de 62 anos e um aneurisma de artéria renal de 2,2 cm de diâmetro em um paciente de 89 anos, ambos do sexo masculino.

Entre os 27 aneurismas incidentalmente detectados, em 5 (18,5%) o tratamento estaria indicado se considerado apenas o diâmetro: um AAA, um aneurisma de ilíaca interna, dois de ilíaca comum e um da artéria renal.

Apesar de a maioria das TCs dessa amostra terem sido realizadas com contraste endovenoso (53,6%), 60% dos aneurismas arteriais foram detectados em TCs sem contraste.

A literatura cita a doença ateromatosa como importante fator de risco na etiologia do aneurisma^3^^,^ 9 ^,^ 10. Entre os aneurismas arteriais detectados nas diferentes topografias, houve uma associação de 80% com ateromatose da aorta.

Este estudo apresenta limitações pelo seu caráter retrospectivo, principalmente devido aos laudos tomográficos descreverem como “ectasias” as dilatações que deveriam ter sido classificadas como “aneurismas”. A utilização de termos inespecíficos, como “dilatações”, também dificultou a interpretação dos laudos; por isso, houve a necessidade de reavaliar os achados, classificando o diâmetro da dilatação detectada de acordo com as referências da literatura. A revisão das imagens correspondentes aos laudos tomográficos randomizados não foi possível, pois apenas os laudos permaneceram arquivados.

## CONCLUSÕES

A prevalência do diagnóstico incidental de aneurismas arteriais nesta amostra foi de 2,2%, sendo 0,6% a prevalência de AAA, 1,1% de AAT, 0,16% de aorta na transição toracoabdominal (ATA), 0,16% de ilíaca comum, 0,08% de ilíaca interna, 0,08% de artéria esplênica e 0,08% de artéria renal.

Em 4,4% das 443 TCs de tórax, houve diagnóstico incidental de aneurismas.

A maioria dos aneurismas acometeu o gênero masculino (60%), e a idade média foi de 81,5 anos.

O diagnóstico incidental de aneurismas foi mais comum na aorta torácica (segmento ascendente) e na aorta abdominal. Os diâmetros médios encontrados em cada segmento arterial da nossa pesquisa foram: 4 cm no AAA, 4,1 cm no AAT e 3,9 cm no ATA.

Os sintomas respiratórios foram a principal indicação para a realização de tomografia computadorizada associada ao diagnóstico incidental de aneurismas.
