# Supplementary material for: Achados incidentais de aneurismas torácicos e abdominais
Source: J Vasc Bras. 2016 Apr-Jun;15(2):106–12. [Article in Portuguese] doi: 10.1590/1677-5449.007616 (PMC5829703; doi:10.1590/1677-5449.007616)
Supplement: Translation

# Translation: Thoracic and abdominal aneurysms as incidental findings

**Authors:** Adenauer Marinho de Oliveira Góes, Bárbara Íris Mascarenhas, Sofia Cunha Rodrigues, Mariseth Carvalho de Andrade, Reinaldo Sergio Monteiro Franco

**Affiliations:** 1 Universidade Federal do Pará – UFPA, Faculdade de Medicina, Belém, PA, Brazil.; 2 Centro Universitário do Estado do Pará – CESUPA, Belém, PA, Brazil.; 3 Universidade do Estado do Pará – UEPA, Belém, PA, Brazil.

**Keywords:** aneurysm, pathological dilation, ectasia, incidental findings

## Abstract

**Background:**

Abdominal aortic aneurysms (AAAs) are the most common. The annual incidence of AAA rupture is eight cases per 100,000 inhabitants. Incidental detection can benefit patients if diameter is monitored and the correct treatment provided.

**Objectives:**

To estimate the prevalence of incidental diagnosis of thoracic aortic aneurysm (TAA) and AAA by computed tomography (CT); to determine prevalence by age and sex of patients and determine which arteries are involved and the morphological characteristics of the aneurysms; to determine the indications for CT most associated with incidental diagnosis of aneurysms.

**Methods:**

This was a descriptive, retrospective and randomized study. Inclusion criteria: patients over 50 years of age examined with CT of the thorax, abdomen or pelvis. Exclusion criteria: follow-up or diagnostic suspicion of aneurysms. Data collection instruments containing questions covering demographic and anatomic data were used.

**Results:**

A sample of 1,202 radiology reports was reviewed. A total of 27 aneurysms were detected (prevalence of 2.2%). Patients: 60% were male and 40% were female (p < 0.05). Sites: there were 13 cases (48.2%) in the ascending aorta (TAA); 7 (25.9%) in the infrarenal aorta (AAA); 2 (7.4%) at the thoracoabdominal transition of the aorta (TTA); 2 (7.4%) in the common iliac artery ; 1 (3.7%) in the internal iliac artery; 1 (3.7%) in the splenic artery; and 1 (3.7%) in the renal artery.

**Conclusions:**

The majority of patients were male (60%); TAA was the most common type (mean diameter: 4.1 cm), followed by AAA (mean diameter: 4.0 cm) and ATA (mean diameter: 3.9 cm). The most common indication for the CT examination that led to incidental diagnosis of an aneurysm was respiratory symptoms.

## INTRODUCTION

Aneurysms are defined as dilation of an artery by more than 50% of the normal diameter of the segment in question.^1^^-^ 20 According to the literature, they are most common in the aorta, followed by the iliac arteries and then the splenic artery.^7^ They have multifactorial etiology (hereditary, traumatic, infectious, and/or inflammatory), but around 80% of aneurysms are associated with atherosclerotic degeneration of the artery wall.^2^^,^ 5

It is estimated that the incidence rate of thoracic aortic aneurysms (TAA) is six cases per 100,000 patients/year, and that the incidence of abdominal aortic aneurysms (AAA) is 25 per 100,000 patients/year.^3^^,^ 5 Around 10% of patients with AAA have aneurysms in another segment of the aorta, and 25% of patients with TAA also have a concomitant AAA.^3^^,^ 7 Elective treatment is recommended to repair a TAA if it is symptomatic or reaches 6 cm in diameter.^1^^,^ 8 ^,^ 20

As life expectancy has increased, diagnoses of AAA have become more common; within the population over 65 years of age, the rate is as high as 6%, and over 80 years of age it reaches 10%.^21^ Diagnoses peak between 65 and 75 years of age.^2^^,^ 9 ^,^ 10 ^,^ 22 The majority of studies define an infrarenal AAA as a dilation with a cross-sectional diameter greater than or equal to 3 cm.^9^^,^ 11 ^,^ 12 ^,^ 19 ^,^ 20 ^,^ 23

Elective treatment is recommended for an AAA when its diameter reaches 5.5 cm or when there is an increase of at least 5 mm in1 year, because of the increased risk of rupture and the potential lethality.

Aneurysms of the iliac arteries in isolation (without aortic involvement) are rare. Common and external iliac arteries with cross-sectional diameters equal to or greater than 1.5 cm are defined as aneurysmal. Elective repair is indicated for aneurysms that are symptomatic and/or for those that are larger than 2.5 cm.^9^^,^ 12 ^,^ 23 ^,^ 24 Aneurysms of the internal iliac artery should be treated irrespective of diameter.^20^

Aneurysms of the splenic artery are the third most common intra-abdominal aneurysms and the most common visceral aneurysms (46 to 60%); they are more common among women (75 to 87%) aged 50 to 79 years.^25^^,^ 26 Indications for treatment include diameter greater than 2 cm, woman of fertile age, pregnancy before the third trimester, if associated with pancreatitis or pancreatic pseudocyst or in symptomatic cases.^20^^,^ 21 ^,^ 25 ^-^ 28

Aneurysms exhibit varying clinical manifestations. They are often asymptomatic and may be detected incidentally or by screening programs.^3^^,^ 4 ^,^ 14 ^,^ 15 ^,^ 29

Incidental diagnosis is an unexpected finding in a radiology examination conducted for some other reason and occurs in 5 to 20% of radiological examinations.^30^^,^ 31 Incidental diagnosis of aneurysms has become more common because of the increase in life expectancy of populations and because of refinements to diagnostic methods and easier access to them.^5^^,^ 9 ^,^ 16 ^,^ 31

It is estimated that the annual incidence of AAA rupture is eight cases per 100,000 inhabitants and that these events are responsible for 2% of deaths in the population over the age of 60.^9^

Since elective treatment to repair aneurysms has lower morbidity and mortality; incidental diagnosis and screening programs contribute to reducing mortality and complications associated with aneurysmal disease.^9^^,^ 13 ^,^ 29 ^,^ 31 ^,^ 32

## OBJECTIVES

General objective:

- To estimate the prevalence of incidental diagnosis of TAAs and AAAs at a private radiology service.

Specific objectives:

To determine:

- Age and sex of patients with incidental diagnoses of TAAs and AAAs.
- The arteries involved and the morphological characteristics of aneurysms diagnosed incidentally.
- The indications for computed tomography that are most associated with incidental diagnosis of TAAs and AAAs.

## METHODS

This was a cross-sectional, descriptive, retrospective, and randomized study for which the sample size was calculated. Data were collected from reports on computed tomography (CTs) examinations of pelvis, abdomen and thorax conducted at a private service from January 2009 to December 2013. The study was approved by the institution’s Research Ethics Committee and registered on the Plataforma Brasil database.

Data collection instruments were used to collect information on: sex and age of patient, date of examination, use of intravenous iodinated contrast, and diameter, length, location and format (saccular or fusiform) of aneurysms. The randomized sample of CT reports was searched for the words “aneurysm,” “dilation,” “dissection” and “ectasia.”

Dilations of the aorta were considered aneurysms if their diameter was greater than or equal to 3 cm and dilated iliac arteries were defined as aneurysmal if their diameter was greater than or equal to 1.5 cm, in accordance with methodologies that have been employed in prior work reported in the literature.^9^^,^ 11 ^,^ 12 ^,^ 23 Smaller dilations were classified as ectasias. Dilations detected in other arteries were classified according to descriptions in the literature.

The sample size calculation was conducted using a formula based on the normal distribution, with a 95% confidence interval. Randomization was performed using BioEstat 5.0.

Inclusion criteria were: CT scans of the thorax, abdomen and/or pelvis of patients aged 50 years or over conducted between January 2009 and December 2013. Exclusion criteria: CT requested because of a diagnostic suspicion of aneurysm or to monitor aneurysms diagnosed previously.

The G test of goodness of fit and the chi-square (χ2) test were used. The significance level adopted was α = 0.05 (5%).

## RESULTS

From January 2009 to December 2013, 3,325 CTs of the thorax, 3,132 CTs of the abdomen and 692 CTs of the pelvis were conducted in patients who met the inclusion criteria for the study. After calculation of the sample size and randomization of the reports, a total of 1,202 radiological reports were selected for analysis.

The reports reviewed broke down as 343 (28.5%) CTs of the thorax, 612 (51%) of the abdomen, and 247 (20.5%) of the pelvis. Reports on abdominal CTs were the most frequent in the sample, with statistical significance (p < 0.0001, χ2 test) (Table 1).

**Table 1:** Distribution of computed tomographies by topography.

Topography	Frequency	%
Abdomen*	612	51%
Pelvis	247	20.5%
Thorax	343	28.5%
TOTAL	1,202	100%

* p < 0.0001 (chi-square).

Table 2 shows the results for use of intravenous iodinated contrast by CT topography. For examinations of the thorax, scans without contrast were statistically more frequent; for the abdomen, scans with contrast were more common (p < 0.0001, χ2 test); and for the pelvis, there was no significant difference.

**Table 2:** Distribution of computed tomographies by topography and use of iodinated intravenous contrast.

**Topography**	**Contrast**				***p-value***
	**YES**	**%**	**NO**	**%**	
Abdomen	415	67.8%	197	32.2%	< 0.0001*
Pelvis	127	51.4%	120	48.6%	0.6554
Thorax	102	29.7%	241	70.3%	< 0.0001*
TOTAL	644	53.6%	558	46.4%	------

* p < 0.05.

The ages of the patients examined varied from 50 to 96 years, with a mean of 66.4 years. Sex distribution of the patients was as follows: 729 (60.6%) examinations were conducted with female patients and 473 (39.4%) with males (p < 0.05, χ2 test) (Figures 1 and 2).

**Figure 1:** Distribution of sample by age group. Source: Database used for study.

**Figure 2:** Distribution of sample by sex. *p < 0.05.

There was no statistically significant difference in sex distribution of patients by topography examined (p = 0.4674, χ2 test) (Figure 3).

**Figure 3:** Distribution by topography of CT examination and sex of patients.

Analysis of the topography examined with CT by age of patients revealed that the age group 50 to 59 years was statistically more common among patients who underwent abdominal CT; while there was a statistically significant predominance of people aged 60 to 69 among patients who underwent CTs of the thorax and the pelvis (p < 0.0001, χ2 test) (Table 3).

**Table 3:** Distribution of computed tomographies by topography and age group of patients.

**Age group (in years)**	**Topography**					
	**Abdomen**	**%**	**Pelvis**	**%**	**Thorax**	**%**
50 to 59	214*	35%*	84	34%	84	24.5%
60 to 69	181	29.6%	90*	36.4%*	120*	35%*
70 to 79	141	23%	52	21.1%	85	24.8%
80 to 89	68	11.1%	19	7.7%	47	13.7%
≥ 90	8	1.3%	2	0.8%	7	2%
TOTAL	612	51%	247	20.5%	343	28.5%
*p*-value	< 0.0001*		< 0.0001*		0.0010*	

* p < 0.0001* (chi-square).

There were 27 aneurysms diagnosed incidentally; 15 (55.5%) out of the 343 thoracic CTs, 7 (26%) out of the 612 abdominal CTs and 5 (18.5%) out of the 247 pelvic CTs. Incidental aneurysm diagnoses were statistically more frequent in thoracic CTs than in the other two topographies (p = 0.0446, χ2 test) (Table 4).

**Table 4:** Distribution of incidentally diagnosed aneurysms according to topography of CT.

**TOPOGRAPHY**	**n**	**Findings**	**%**
Thorax	343	15*	4.4%*
Pelvis	247	5	2%
Abdomen	612	7	1.1%

* p = 0.0446 (chi-square).

The 27 aneurysms were found in 25 patients (two male patients each had two aneurysms simultaneously). Table 5 shows the distribution of aneurysms according to artery involved, mean diameter of aneurysm and sex and mean age of patients. Male patients were statistically more frequent among those with aneurysms of the infrarenal aorta (p = 0.0116, G test of goodness of fit).

**Table 5:** Distribution of aneurysms by site, sex and age of patient and mean diameter.

**Site**	**n**	**FEMALE**		**MALE**		**Mean age**	**Mean diameter**	**G test of goodness of fit,** ***p*-value**
Ascending aorta	13	7	53.8%	6	46.2%	71.9	4.1	0.844
Aorta at thoracoabdominal transition	2	1	50%	1	50%	72.5	3.9	N/A
Infrarenal abdominal aorta	7	2	28.6%	5	71.4%*	77	4	0.0116*
Right common iliac artery	1	0	0%	1	100%	66	2.6	N/A
Left common iliac artery	1	0	0%	1	100%	76	3	N/A
Left internal iliac artery	1	0	0%	1	100%	85	1.7	N/A
Left renal artery	1	0	0%	1	100%	89	2.2	N/A
Splenic artery	1	0	0%	1	100%	62	1.2	N/A

* p < 0.05 (teste G de aderência).

N/A = not applicable.

Analysis of the indications for CT showed that indications for respiratory symptoms were the most frequent, with statistical significance (p = 0.0001, G test of goodness of fit), and accounted for 10 cases. The other indications are summarized in Table 6.

**Table 6:** Distribution by indication for computed tomography in patients with incidental diagnosis of aneurysms.

**Indications**	**Frequency**	**%**
Respiratory symptoms	10*	37%*
Genitourinary symptoms	5	18.5%
Gastrointestinal symptoms	5	18.5%
General symptoms	3	11.1%
Liver tumor	2	7.4%
Not stated	2	7.4%
TOTAL	27	92.6%

* p = 0.0001 (G test of goodness of fit).

Other findings worthy of note were seven arterial ectasias, most frequently involving the abdominal aorta, and the fact that 20 of the 25 patients diagnosed with arterial aneurysms had atheromatous plaques in the aorta.

## DISCUSSION

References in the literature on incidental diagnosis of aneurysms habitually focus on a specific artery; the abdominal aorta in the majority of cases. This study differs from others in that it investigates incidental diagnosis of aneurysms involving any thoracic and/or abdominal artery; although the results have been stratified in a manner that allows comparison with the literature.

The predominance of aneurysmal disease among men has already been described by many authors.^1^^-^ 17 ^,^ 22 ^,^ 23 ^,^ 30 ^-^ 32 While the 60.6% of the sample were CTs of female patients, there was a predominance (although not a statistically significant difference) of diagnoses among males [15 (60%) men and 10 (40%) women].

According to the literature, AAA diagnoses peak between 65 and 75 years of age.^9^^,^ 11 ^,^ 12 ^,^ 23 The average age of patients with AAA in the present sample was 77 years, similar to data reported by Barros et al. in a study of screening for AAA in a population from Vitória, where mean age was 74.1 years^16^; and older than the age observed by Silva et al., who reported mean age of 69.4 years.^10^

The overall prevalence of arterial aneurysms was 2.2%. The prevalence of AAA was 0.6% for both sexes and 1% for men only. The prevalence of AAA observed was lower than rates reported by Molnar et al. (2.1%),^12^ Barros et al. (2.5%),^16^ Bonamingo and Siqueira (3.7%),^11^ and Silva et al. (4.49%).^10^ A diagnosis of AAA was more frequent among male patients (71.4%), in agreement with the literature.^1^^-^ 9 ^,^ 17 ^,^ 22 ^,^ 30 ^-^ 32

The mean diameter of the AAAs was 4 cm (range: 3.1 cm to 6.9 cm), which is similar to two other Brazilian studies, which found mean diameters of 3.9 cm^9^ and 3.5 cm.^16^

Elective treatment of AAAs is recommended when their diameter reaches 5.5 cm.^4^^,^ 11 ^,^ 13 ^,^ 32 One of the incidentally diagnosed AAAs had a diameter of 6.9 cm. We highlight this finding because it is estimated that the 5-year risk of rupture of aneurysms with diameters smaller than 5 cm is less than 5%, whereas diameters greater than 5 cm involve risk of 25 to 43%.^1^^,^ 3

The prevalence of TAAs was 0.99%. The 13 TAAs involved the ascending aorta, the segment that has previously been described as most often involved in thoracic aorta aneurysms.^3^ Progressive dilation of the ascending aorta can lead to aortic valve insufficiency, acute dissection or spontaneous rupture,^1^ all of which are events that dramatically alter patient survival.

Two aneurysms were also found at the thoracoabdominal transition of the aorta (TTA), accounting for 7.7% of all of the aneurysms and equating to a prevalence of 0.16% for the whole sample. We did not find any studies about incidental diagnosis of aneurysms in this topography. However, there are references stating that aneurysms of the thoracoabdominal transition account for 10.6^3^ to 31.6%^5^ of all aortic aneurysms.

In the present study, TAAs predominated, accounting for almost half of all of the aneurysms found, even though the majority of the CT reports analyzed were abdominal (50.9%). This results differs from previous research that has found that the infrarenal aorta is the segment of the aorta most often affected by aneurysms.^1^^,^ 3 ^,^ 8 ^,^ 10 ^,^ 13

The mean diameter of TAAs was 4.1 cm; however, the lack of literature on the subject means that this result cannot be compared and the same is true of the mean age of patients with TAA. None of these patients had an aneurysm with diameter greater than or equal to 6 cm, which is the cutoff beyond which surgical intervention is generally indicated.^1^^,^ 8

Aneurysms of the common iliac artery are candidates for elective surgery when their diameters exceed 2.5 cm,^12^^,^ 23 ^,^ 24 whereas repair of internal iliac aneurysms is indicated at any diameter.^20^ Two aneurysms of the common iliac artery were found, one with a diameter of 3.0 cm and the other with a diameter of 2.6 cm. One aneurysm of an internal iliac artery was found, measuring 1.7 cm.

According to references, 85% of aneurysms of the common iliac artery are associated with an AAA; while this combination occurs in 10% of aneurysms of internal iliac arteries and 1% of aneurysms of external iliac arteries.^24^ The only case of internal iliac artery aneurysm detected in our sample also had a concomitant AAA. While the literature describes the combination of an aneurysm of the common iliac artery with an aneurysm of the infrarenal aorta as frequent,^20^^,^ 23 ^,^ 24 ^,^ 33 the cases of common iliac aneurysm observed in this study both occurred in isolation.

According to the literature, visceral aneurysms predominate in females and the indications for surgical treatment are as follows: diameter greater than or equal to 2 cm, rupture, symptoms, rapidly progressive growth, particularly in pregnant women or women of fertile age.^20^^,^ 25 The most common visceral aneurysms are: splenic artery aneurysms (46 to 60%), aneurysms of renal arteries (17 to 30%), hepatic artery aneurysms (9 to 20%), superior mesenteric aneurysms (3 to 5%) and celiac trunk aneurysms (3 to 4%).^20^^,^ 25 In the sample analyzed here, one aneurysm of the splenic artery was detected, with a diameter of 1.2 cm, in a 62-year-old patient, and one aneurysm of the renal artery, with a 2.2 cm diameter, in an 89-year-old patient, both male.

A total of 27 aneurysms were detected as incidental findings and 5 (18.5%) of them had indications for treatment based on diameter alone: one AAA, one aneurysm of the internal iliac artery, two of the common iliac artery and one of a renal artery.

Although the majority of CT examinations in the sample were conducted with intravenous contrast (53.6%), 60% of the arterial aneurysms were detected in CTs without use of contrast.

The literature cites atheromatous disease as an important risk factor in the etiology of aneurysms_._ 3 ^,^ 9 ^,^ 10 In this sample, 80% of the arterial aneurysms detected in all the different topographies had an association with atheromatosis of the aorta.

This study has limitations related to its retrospective character, primarily related to the CT reports describing dilations that should have been classified as “aneurysms” as “ectasias.” Use of nonspecific terms such as “dilations” also made it more difficult to interpret the reports and made it necessary to reassess the findings, classifying the diameter of dilation detected according to references in the literature. It was not possible to review the images corresponding to the randomized CT reports because only the reports are archived.

## CONCLUSIONS

The prevalence of incidental diagnosis of arterial aneurysm was 2.2% in this sample, with prevalence rates of 0.6% for AAA, 1.1% for TAA, 0.16% for TTA, 0.16% for common iliac artery aneurysm, 0.08% for internal iliac artery aneurysm, 0.08% for splenic artery aneurysm, and 0.08% for renal artery aneurysm.

There was an incidental diagnosis of aneurysm in 4.4% of the 443 thoracic CTs.

The majority of the aneurysms were in males (60%) and the average age was 81.5 years.

Incidental diagnosis of aneurysm was most common in the thoracic aorta (ascending segment) and the abdominal aorta. The mean diameters observed in each arterial segment were: 4 cm for AAA, 4.1 cm for TAA and 3.9 cm for TTA.

Respiratory symptoms were the most common indication for conducting the computed tomography leading to incidental diagnosis of aneurysms.
